# Effect of Light Modification by Shading Nets on Yield, Composition, and Antioxidant Activity of *Lavandula angustifolia* Mill. Essential Oil

**DOI:** 10.3390/plants15030377

**Published:** 2026-01-26

**Authors:** Zoran S. Ilić, Lidija Milenković, Ljiljana Stanojević, Aleksandra Milenković, Ljubomir Šunić, Bratislav Ćirković, Dragan Božović, Dragan Cvetković, Jelena Stanojević

**Affiliations:** 1 Faculty of Agriculture, University of Priština in Kosovska Mitrovica, 38219 Lesak, Serbia; lidija.milenkovic@pr.ac.rs (L.M.); ljubomir.sunic@pr.ac.rs (L.Š.); bratislav.cirkovic@pr.ac.rs (B.Ć.); 2 Faculty of Technology, University of Niš, Bulevar Oslobodenja 124, 16000 Leskovac, Serbia; ljiljas76@yahoo.com (L.S.); aleksandra.milenkovic@student.ni.ac.rs (A.M.); dragancvetkovic1977@yahoo.com (D.C.); jelena_stanojevic@yahoo.com (J.S.); 3 Research and Development Institute-Tamiš, Novoseljanski Put 33, 26000 Pancevo, Serbia; draganbozovic64@gmail.com

**Keywords:** color shade nets, lavender, essential oil, components, antioxidant activity

## Abstract

In the present study, the yield, chemical composition, and biological activities of *Lavandula angustifolia* flower essential oil (LAFEO) and leaves (LALEO) under different shade nets (pearl, red, blue) with 40% shading index compared with non-shading (control-open field) plants were investigated. The essential oil (EO) was isolated using a Clevenger-type hydrodistillation and the chemical composition of isolated EO was determined by GC/MS and GC/FID analyses. The antioxidant activity was determined using the DPPH and FRAP assay. The highest EO yield was recorded in flowers from plants grown under pearl shade nets (4.62 mL/100 g p.m.) and in leaves under red nets (0.99 mL/100 g p.m.). The lowest EO content occurred in plant leaves (0.50 mL/100 g p.m.) and flowers (3.17 mL/100 g p.m.) from non-shaded (control) plants. The composition of lavender EO depended on both plant part and light conditions. Among the 47–59 identified compounds in LAFEO, the major constituents were 1,8-cineole (27.4–32.2%), linalool (24.7–27.3%), borneol (18.0–21.9%), and camphor (7.5–8.6%). In LALEO, 55–65 compounds were identified, with 1,8-cineole (30.4–39.8%), borneol (21.9–26.5%), camphor (11.3–13.9%), and linalool (6.0–8.6%) as the dominant constituents. Flower samples from non-shaded (control) plants showed moderate antioxidant activity, with EC_50_ values decreasing over time, indicating the highest activity among treatments tested. Conversely, plant leaves under pearl nets showed the lowest activity among samples, with an EC_50_ value of 42.40 mg/mL at 120 min, still within the moderate antioxidant activity range. LALEO showed higher FRAP values than flower oils, confirming a stronger reducing capacity. The highest activity was found in plant leaves under red nets (0.72 mg EFe^2+^/g) and in non-shaded plants (0.68 mg EFe^2+^/g), while the lowest occurred in flower samples from red (0.28 mg EFe^2+^/g) and pearl nets (0.33 mg EFe^2+^/g). Unlike the FRAP results, the DPPH assay showed relatively higher activity in flowers compared to leaves, though all samples exhibited moderate antioxidant capacity. Shading significantly increased essential oil yield; however, the effects of different color nets on essential oil quality require further investigation, although preliminary results indicate a potential reduction in undesirable constituents.

## 1. Introduction

Light quantity and quality regulates the whole life cycle of plants through light receptor conduction, and the morphological structure, photosynthesis and organ growth, and development of plants will have different effects under different light quality [1,2,3]. Manipulating a plant’s light spectrum can cause specific physiological responses, such as changes in secondary metabolite production and biomass allocation, while diffuse light improves light penetration into the inner canopy, helping lower leaves receive more light for photosynthesis [4]. Shading of plants results in numerous changes in the microclimate as well as in plant activity. These microclimatic changes relate to alterations in CO_2_ levels and assimilation, and consequently influence plant growth and development.

Depending on their color and weave density (shade index), these nets provide a combination of natural, unaltered light along with spectrally modified, diffused light. Besides offering physical protection (from hail, strong winds, sandstorms, and airborne pests such as birds, bats, and insects that may transmit viral diseases), they are designed to optimize the desired physiological effects on plants [5]. The production of photo-selective nets relies on incorporating various chromatic additives, as well as components that enhance light dispersion and reflection within the material. Their structure enables selective transmission of different spectral components of solar radiation (UV, visible, and long wavelengths) and/or direct transformation of light into a diffuse, scattered form. Thus, black, gray, and white nets reduce the light quantity (neutral shade), while red, blue, yellow, and pearl nets change the red and blue light composition (photo-selective shade) [6].

Light modification by colored shading nets shows promise in modulating EO content and composition in different aromatic and medicinal plants [7,8]. Their effectiveness is highly dependent on plant species, genotype-specific traits, and environmental conditions [9]. Shading plants by photo-selective shade nets synthesized more EOs than plants exposed to full sunlight [10]. Marjoram and oregano tolerate shading well and gave higher essential oil yield when cultivated under shade. Lemon balm, mint, and sweet basil [7], or sage, oregano, and rosemary [10] produce higher essential oil content under shaded conditions. Sage and basil plants covered by blue shade nets produce the highest essential oils yield and antioxidant activity by a significant margin in comparison with other nets and non-shading plants [11].

*Lavandula angustifolia* Mill., lavender, originates from the Mediterranean region and belongs to the Lamiaceae family, which is well-known for their evergreen medicinal plants whose oil is recognized for its exceptional fragrance. This aromatic plant species is economically important and is used in cosmetics, aromatherapy, food, and medicine [12]. Lavender EO has antifungal activities, which supports their promising fungicidal potential [13]. In addition to lavender volatiles, there are other active ingredients such as flavonoids, coumarin, tannins, and phenolic compounds; the main feature of herbal flavonoids and phenolic compounds is their antioxidant activity, which has led to many drug applications [14]. The chemical composition of the essential oil (EO) depends on a number of parameters, such as the environmental conditions, the drying procedure, the storage conditions, the method of isolation of the essential oil, and the analysis conditions, which are used for the identification of the compounds [15]. Fresh lavender flowers contain 0.5–6.25% essential oil [16], though some are lost during drying [17]. In dry inflorescences, lavender essential oil content ranges from 0.5% to 9.62% [16]. For *Lavandula × intermedia* cv. ‘Budrovka’ (Serbia, Fruška Gora Mt.), oil content dropped from 1.03% to 1.26% [18], below the 1.3% minimum set by the European Pharmacopoeia [19]. The optimization of lavender flower pretreatment methods before hydrodistillation affects essential oil yield [20]. Different plant parts are characterized by different EO content and composition. The predominant compounds in the oil obtained from leaves were epi-α-cadinol (17.8%), cryptone (10.4%), 1,8-cineole (7.3%), and caryo-phyllene oxide (7.2%), and of the oil distilled from flowers, linalyl acetate (22.3–32.1%) and linalool (23.9–29.9%) [21].

Considering climate change and its negative effects on the cultivation of many plant species, the aim of this study was to examine the influence of protecting lavender plants with colored shading nets and how they affect the yield and quality of essential oils in different plant parts of lavender. The objective was also to assess whether the quality of the oil complies with the standard requirements for lavender essential oil composition, as well as with available literature data.

## 2. Material and Methods

### 2.1. Plant Material

The experiment with cultivated *Lavandula angustifolia* Mill. (lavender) was conducted in 2024 in an experimental garden in the village of Moravac in south Serbia (21°42′ E, 43°30′ N, altitude 159 m a.s.l.) Lavender seedlings were planted in early May at approximately two years of age, bearing around three primary branches and reaching 15–18 cm in height. Planting was conducted in prepared, fertilized soil (Elixir Zorka Supreme 12:11:18 + 2% MgO + 15% S + 0.01% B + 0.02% Zn + TE).

It was a monofactorial experiment. Each experimental plot measured 5 m in length and 2.4 m in width (12 m^2^). Each plot consisted of three rows, and the spacing between the rows was 90 × 40 cm (2.8 plants/m^2^). A 1 m distance was provided between the plots. Ten plants from the central rows from all samples were selected as a representative sample for all analyses.

#### 2.1.1. Irrigation Management

Due to severe drought in July and August, drip irrigation was applied. The study employed a drip irrigation system, utilizing 16 mm lateral polyethylene pipes with in-line emitters spaced 50 cm apart, delivering water at a rate of 2 L h^−1^ under a pressure of 1 atm.

#### 2.1.2. Environmental Monitoring

To protect plants from excessive temperature and solar radiation during summer, colored shading nets (pearl, red, and blue; Polysack, Nir Yitzhak D.N. NegevIsrael) with a 40% shading index were installed horizontally 2 m above the canopy. These nets selectively modify solar radiation by altering spectral composition and increasing light diffusion.

Photosynthetically active radiation (PAR) above the canopy was measured using a ceptometer (SunScan SS1, Delta-T Devices, Cambridge, UK) and expressed as PAR quantum flux (µmol m^−2^ s^−1^). Measurements were taken several time points during the day (from 6 h to 18 h) under cloudless conditions, repeated weekly throughout the growing season (June–September). Solar irradiation was recorded using a portable solarimeter (SL 100, KIMO, Montpon, France; 1–1300 W m^−2^).

Air temperature and relative humidity and light (PAR) under each shading net and in the open field were monitored using data loggers (Spectrum Technologies Inc. Wachdog model 2475 Plant Growth Station, Chicago, IL, USA), positioned at canopy height, recording at 60 min intervals throughout the experimental period.

#### 2.1.3. Harvest and Yield Determination

Flowering stems developed in late June and were harvested at full bloom stage. Inflorescences (spike-like structures) were cut and immediately weighed to determine fresh yield. Samples were then air-dried in a shaded, well-ventilated area, and dry weight was recorded to calculate fresh-to-dry mass ratios. In September, after formation of additional branches, green biomass (leaves and stems) was harvested once per plot. At this stage, the vegetative biomass of the plants was sufficiently developed to withstand cutting. The apical parts of the branches, about 10 cm in length, were cut. All yields were calculated and expressed as tonnes per hectare (t/ha) based on plot dimensions and planting density.

### 2.2. Reagents and Chemicals

Ethanol 96% p.a. (Reahem d.o.o., Novi Sad, Serbia), 2,2-diphenyl-1-picrylhydrazyl (DPPH) radical, (2,2′-azinobis(3-ethylbenzothiazoline-6-sulfonic acid (ABTS), 2,4,6-tris(2 pyridyl)-s-triazine (TPTZ), iron (III) chloride hexahydrate (Sigma Chemical Company, St. Louis, MO, USA). All other chemicals are of analytical reagent grade (p.a.).

### 2.3. Clevenger-Hydrodistillation

Disintegrated and homogenized plant material (lavender leaves and lavender flowers) previously air-dried in a shaded, well-ventilated area was used for essential oil isolation by Clevenger-type hydrodistillation, with hydromodule (ratio of plant material/water) 1:15 *m*/*v* during 120 min [22]. During the distillation the volume of separated oil was read in the measuring Clevenger’s tube after 15–120 min, and monitored depending on the yield of the essential oil over the time. The oil isolated from lavender leaves and flowers was separated from the measuring tube after distillation, dried over anhydrous sodium sulfate, and stored in dark bottles in a refrigerator at +4 °C.

### 2.4. Gas Chromatography/Mass Spectrometry (GC/MS) and Gas Chromatography/Flame Ionization Detection (GC/FID) Analysis

GC/MS analysis was performed on an Agilent Technologies 7890B gas chromatograph equipped with a nonpolar silica capillary column, HP-5MS (5% diphenyl- and 95% dimethyl-polysiloxane, 30 m × 0.25 mm, 0.25 μm film thickness; Agilent Technologies, Santa Clara, CA, USA), and coupled with an inert, selective 5977A mass detector of the same company. The samples were dissolved in diethyl ether. In total, 1 μL of the prepared solution was injected into the GC column through a split/splitless inlet set at 220 °C in 40:1 split mode. Helium was used as the carrier gas at a constant flow rate of 1 cm^3^/min. The oven temperature increased from 60 °C to 246 °C at a rate of 3 °C/min. The temperatures of the MSD transfer line, ion source, and quadrupole mass analyzer were set at 300 °C, 230 °C, and 150 °C, respectively. The ionization voltage was 70 eV, and the mass range was *m*/*z* 41–415.

GC/FID analysis was carried out under identical experimental conditions as GC/MS. The flows of the carrier gas (He), make up gas (N_2_), fuel gas (H_2_), and oxidizing gas (Air) were 1 cm^3^/min, 25 cm^3^/min, 30 cm^3^/min, and 400 cm^3^/min, respectively. The temperature of the flame-ionization detector (FID) was set at 300 °C.

Data processing was performed using MSD ChemStation Data Analysis (version F.01.00.1903), AMDIS (Automatic Mass Spectral Deconvolution and Identification System, version 2.70), and NIST MS Search (version 2.0 g) software (Agilent Technologies, Santa Clara, CA, USA). Retention indices of the components from the analyzed samples were experimentally determined using a homologous series of *n*-alkanes from C_8_–C_20_ as standards. Constituent identification was based on the comparison of their retention indices (RI^exp^) with those available in the literature (RI^lit^) and their mass spectra (MS) with those from Willey 6, NIST2011, and RTLPEST3 libraries. Semi-quantitative analysis was performed using the area normalization method of the GC/FID signal without corrections.

### 2.5. DPPH Assay

The ability of the essential oil to scavenge free DPPH radicals was determined using the DPPH assay. Essential oils were dissolved in the ethanol, and a series of different concentrations was prepared (25–0.781 mg/mL). Ethanol solution of DPPH radical (1 mL, 300 μmol solution (3 × 10^−4^ mol/dm^3^)) was added to 2.5 mL of the prepared essential oil solutions. Absorption was measured at 517 nm after 20, 60, and 120 min incubation with DPPH radical. Absorption at 517 nm was determined for the ethanolic solution of DPPH radical as well, which was diluted in the aforementioned ratio (1 mL of the DPPH radical of the given concentration with 2.5 mL ethanol added). Ethanol was used as a blank. Free radical scavenging activity was calculated according to the formula [23]

$$\mathrm{DPPH}\;\mathrm{radical}\;\mathrm{scavenging}\;\mathrm{capacity}\;(\%)=100-\left[\left(A_{S}-A_{B}\right)\times \frac{100}{A_{C}}\right]$$

*A*_S_—Absorption of the “sample” at 517 nm. “Sample”—ethanolic solution of the essential oil treated with DPPH radical solution.

*A*_B_—Absorption of the “blank” at 517 nm. “Blank”—ethanolic solution of the essential oil which is not treated with DPPH radical solution

*A*_C_—Absorption of the “control” at 517 nm. “Control”—ethanolic solution of the DPPH radical

All absorptions were measured on Perkin Elmer Lambda 25, Spectrophotometer (Shelton, CT, USA).

The essential oil concentration needed for the neutralization of 50% of the initial DPPH radical concentration is called EC_50_ value. This value was determined by using linear regression analysis of the range of different concentrations of essential oil added to the reaction mixture.

#### FRAP (Ferric Reducing Ability of Plasma) Assay

The antioxidant activity of lavender essential oil was determined using the FRAP test according to the method of Benzie and Strain [24], with certain modifications according to Stanojević et al. [25].

The procedure for determining the antioxidant activity of extracts was as follows: The 0.1 cm^3^ of ethanolic solutions of lavender essential oil were added to tubes, followed by 3 cm^3^ of FRAP reagent to each tube. After incubation for 30 min (at 37 °C in a water bath), the absorbance was measured at 593 nm, in relation to the blank sample (3 cm^3^ of FRAP reagent + 0.1 cm^3^ of 96% *v*/*v* ethanol).

The concentration (mmol/dm^3^) of Fe^2+^ equivalents in each sample was read directly from the FeSO_4_ × 7H_2_O calibration curve (A_593nm_ = 0.03521 + 0.5949·*c* FeSO_4_ × 7H_2_O (mmol/L), R^2^ = 0.993) [26]. The results were expressed as concentration of Fe^2+^ equivalents per gram of essential oil (mg EFe^2+^/g e.o.). This value is called the FRAP value.

### 2.6. Statistical Analysis

All measurements were conducted in triplicate, and results were expressed as mean ± standard deviation. For morphological evaluation and biochemical analyses, the data were analyzed using one-way ANOVA, followed by Duncan’s multiple range test (*p* < 0.05).

## 3. Results

### 3.1. Microenvironment Under Different Color Shade Nets

Under nets, photosynthetically active radiation (PAR) and solar radiation were significantly lower (more than 40%) compared to the control (open field condition)—Table 1.

**Figure 1:**
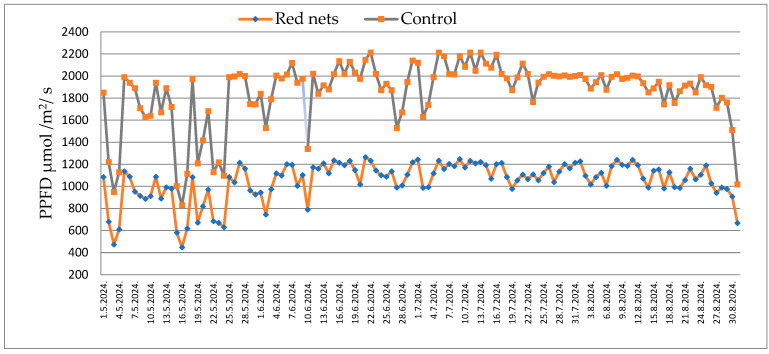
Maximum value of PAR in May, June, July, and August 2024 (control—open field and red shade net). PAR—Photosynthetically active radiation.

During the three hottest summer months (June, July, and August), photosynthetically active radiation (PAR) above the canopy, expressed as PPFD, exceeds 2000 µmol m^−2^ s^−1^ in open field conditions, whereas under red shading nets these values range between 1000 and 1200 µmol m^−2^ s^−1^. In July, shading with pearl, red, and blue nets decreased the mean PPFD by 45%, 41%, and 50%, respectively, compared to the unshaded (open field) control condition. Also, shading substantially reduced light availability compared to the unshaded open field condition control group (996 W/m^2^), with mean irradiance decreased by 40% under the pearl net, 35% under the red net, and 44% under the blue net. PPFD fluctuations during this period are minimal, and the recorded values are comparable to, or even higher than, those typical of the Mediterranean region (Figure 1).

Overall, photo-selective netting represents a cost-effective strategy for manipulating crop microclimate conditions and plant growth, allowing the regulation of not only yield but also quality and functional or bioactive plant properties.

### 3.2. Plant Growth and Biomass Yield

Plant shading and the modification of natural light affect morphological characteristics and the most important yield parameters. Thus, plant height, number of branches/shoots, plant biomass, leaf mass, and inflorescence (flower) mass were significantly higher in plants shaded with pearl and red nets than in those shaded with blue nets and in non-shaded plants grown in the open field (Table 2).

Harvesting of spike-like lavender inflorescences was carried out in late June. As the plants were in their second year of growth, harvesting was performed once, when the majority of flowers were fully open. The yield of fresh inflorescences obtained from plants grown in the open field (0.78 t·ha^−1^) and under the blue net (0.72 t·ha^−1^) was significantly lower compared to the values recorded under the red and pearl nets (1.30–1.32 t·ha^−1^). In addition to their positive effects on the morphological traits of lavender plants, shading with red and pearl nets also positively affected the yield of fresh and dry inflorescences per unit area. Shading resulted in a more uniform fresh-to-dry mass ratio of spike-like inflorescences, regardless of the color of the applied photo-selective nets.

A single harvest of the aboveground vegetative biomass (leaves and stems) of lavender plants was carried out in the last week of September. The fresh biomass yield of plants grown under the blue net (6.10 t·ha^1^) was significantly lower (*p* < 0.01) than that of plants grown under the pearl net (7.90 t·ha^−1^). The positive effect of the red and pearl nets on lavender was even more pronounced when compared with plants grown without shading in the open field. The use of photo-selective nets, especially red and pearl ones, represents an effective agrotechnical approach that enhances both vegetative and generative biomass production in lavender, with potential positive effects on quality. The altered light quality caused by shading affects plant performance, often resulting in higher yields for medicinal plants. However, the specific effects depend on the color and type of the photo-selective net, as these determine both the light spectrum and intensity reaching the canopy.

Crop shading results in numerous changes in the microclimate but also in plant activity. These microclimate changes are related to CO_2_ exchange, assimilation, and thus indirectly to the growth and development of plants and secondary metabolite biosynthesis [26].

### 3.3. Essential Oil (EO) Yield

The EO yield showed clear differences between the flower and leaf samples of *Lavandula angustifolia* Mill. In all cases, flower material showed a significantly higher lavender EO (LAFEO) yield compared to leaves (LALEO). This outcome is expected, as floral tissues contain a higher density of glandular trichomes responsible for essential oil accumulation [27].

Overall, the results confirm that flowers are the most efficient source of lavender essential oil: the highest LAFEO yields were obtained from plants covered by pearl (4.62 mL/100 g) and red shade nets (4.39 mL/100 g), while leaves contribute much smaller amounts, although they may still influence the final aromatic profile. The lavender leaf EO (LALEO) yields remained below 1.00 mL/100 g p.m. Slightly lower LALEO yields were observed in plants shaded by blue nets and non-shaded plants (0.50 ± 0.02 mL/100 g p.m.) from the open field (Table 3).

The composition of the essential oils of lavender depends on the plant part and the light conditions. From the total constituents (47–59), the key constituents in LAFEO were 1,8-Cineole (27.4–32.2%), linalool (24.7–27.3%), borneol (18.0–21.9%), and camphor (7.5–8.6), while in LALEO, we obtained 55–65 different constituents with dominant 1.8-cineole (30.4–39.8%), borneol (21.9–26.5%), camphor (11.3–13.9%), and linalool (6.0–8.6%).

The highest number of total identified components in LAFEO (59) was recorded in plants covered with pearl nets, while the lowest number (47) was observed under blue nets. The most abundant component was 1.8-cineole, ranging from 27.4% in non-shaded plants to 32.2% in plants covered with red nets. The second most abundant component was linalool, accounting for 24.7% in plants grown under red nets and in the control, with the highest content (27.3%) recorded under pearl nets. Borneol was the third most abundant component, ranging from 18.0% in plants shaded with pearl nets to 21.9% in non-shaded plants (Table 4).

Camphor, a component known to reduce essential oil quality, was most abundant in non-shaded plants (8.6%), while the lowest content was recorded in flowers from plants covered with blue nets.

Oxygen-containing monoterpenes were the most dominant group in LAFEO, ranging from 95.3% under pearl nets to 94.5% under red and blue shading nets. Monoterpene hydrocarbons (1.1–1.4%), oxygen-containing sesquiterpenes (0.9–1.2%), and sesquiterpene hydrocarbons (0.2–0.5%), as well as other components (2.5–2.8%), were present to a much lesser extent (Table 4).

The highest number of total identified components in LALEO (65) was recorded in plants covered with red nets, while the lowest number (55) was observed in plants from open field (non-shading). The most abundant component was 1.8-cineole, ranging from 30.4% in plants covered by red nets to 39.8% in plants covered with blue nets. The second most abundant component was borneol, accounting for 21.9% in plants grown under blue nets, with the highest content (26.3%) recorded under red nets. Camphor was the third most abundant component, ranging from 11.3% in plants shaded with blue nets to 13.9% in shaded plants with pearl nets. Linalool was the fourth most abundant in non-shaded plants (8.6%), while the lowest content was recorded in flowers from plants covered with blue nets (6%). Oxygen-containing monoterpenes were the most dominant group in LALEO, ranging from 88.9% under blue nets to 91.3% under pearl shading nets. Monoterpene hydrocarbons (2.5–4.9%), oxygen-containing sesquiterpenes (1.9–3.2%), and sesquiterpene hydrocarbons (0.2–0.5%), as well as other components (3.3–4.1%), were present to a much lesser extent (Table 5).

All EOs were characterized as pleasant, with a floral aroma as a prominent odor.

### 3.4. Antioxidant Activity

The EC_50_ values obtained for all samples show clear differences between essential oils obtained from flowers and leaves, as well as consistent changes over incubation time. In general, lower EC_50_ values indicate higher antioxidant activity, meaning the sample requires a smaller concentration to neutralize 50% of the initial concentration of DPPH radicals.

EC_50_ values decrease progressively from 20 to 120 min, demonstrating that antioxidant activity increases with longer incubation time. Flower samples generally exhibited lower EC_50_ values than leaf samples, indicating relatively higher radical scavenging activity in floral material. Among all samples, flowers from non-shaded (control) plants showed the highest antioxidant activity, with EC_50_ values of 51.97 mg/mL (20 min), 32.26 mg/mL (60 min), and 20.26 mg/mL (120 min). According to standard classification schemes, EC_50_ values between 10 and 50 mg/ mL indicate moderate antioxidant activity, while values > 50 mg/mL indicate weak activity. Therefore, our samples exhibited primarily moderate (20.26–42.40 mg/mL at 120 min) to weak (51.97 mg/mL at 20 min) antioxidant capacity (Table 6).

According to standard classification for DPPH radical scavenging activity [32], EC_50_ values <10 mg/mL indicate strong antioxidant activity, 10–50 mg/mL indicate moderate activity, and >50 mg/mL indicate weak activity. Based on this scale, the lavender essential oils tested in this study exhibited primarily moderate antioxidant activity at 120 min incubation (EC_50_ = 20.26–42.40 mg/mL), with initial weak activity at 20 min (EC_50_ = 51.97–78.58 mg/mL) improving over extended reaction time. For comparison, synthetic antioxidants such as BHT typically show EC_50_ values of 5–15 mg/mL, while natural compounds like ascorbic acid exhibit EC_50_ values of 2–5 mg/mL [32].

The FRAP values obtained for all samples indicate notable differences in their reducing power, expressed as mg Fe^2+^ equivalents per gram of essential oil. In general, higher FRAP values correspond to stronger antioxidant capacity, reflecting the ability of the sample to donate electrons and reduce ferric ions (Fe^3+^) to ferrous ions (Fe^2+^).

Essential oils obtained from leaves showed higher FRAP values than essential oils obtained from flowers, suggesting that leaf-derived oils possess a stronger reducing ability under the conditions of the FRAP assay. The highest activity was observed in leaves (0.72 mg EFe^2+^/g) from red nets and leaves (0.68 mg EFe^2+^/g) from non-shading plants. In contrast, the lowest FRAP activities were recorded in the corresponding flower samples from red (0.28 mg EFe^2+^/g) and pearl nets (0.33 mg EFe^2+^/g). This pattern differs from the DPPH assay results, where flowers generally showed stronger radical-scavenging activity (Table 7).

Such divergence is not uncommon, as FRAP and DPPH measure different antioxidant mechanisms, with FRAP evaluating ferric ion reduction and DPPH measuring hydrogen atom or electron donation to a stable radical. The divergence between DPPH and FRAP results requires mechanistic explanation. While flowers exhibited superior DPPH radical scavenging activity, leaves demonstrated stronger ferric-reducing capacity in the FRAP assay. This apparent contradiction reflects fundamental differences in antioxidant mechanisms and the chemical nature of active compounds in each tissue.

DPPH assay measures single-electron transfer (SET) and hydrogen atom transfer (HAT) mechanisms, favoring compounds with phenolic hydroxyl groups capable of donating hydrogen atoms to stabilize free radicals [33]. The higher linalool content in flowers (24.7–27.3%) compared to leaves (6.0–8.6%) likely contributes to superior DPPH activity, as linalool’s tertiaryhydroxyl group readily donates hydrogen to DPPH radicals [34].

FRAP assays, conversely, exclusively measure electron-donating capacity under acidic conditions (pH 3.6), reflecting the reduction of Fe^3+^-TPTZ complex to Fe^2+^form [35]. The higher FRAP values in leaves may result from (1) elevated camphor content (11.3–13.9% vs. 7.5–8.6% in flowers), whose carbonyl group participates in electron transfer reactions; (2) greater abundance of borneol (21.9–26.5% vs. 18.0–21.9%), a secondary alcohol with stronger reducing properties than tertiary alcohols; and (3) possibly higher concentrations of non-volatile phenolic compounds (e.g., rosmarinic acid, caffeic acid derivatives) that were not detected by GC/MS but are known to be more abundant in photosynthetic leaf tissue and exhibit exceptional FRAP activity [36].

Additionally, 1,8-cineole, the most abundant compound in both flowers (27.4–32.2%) and leaves (30.4–39.8%), is an ether with minimal antioxidant activity in both assays, explaining why total essential oil content does not directly correlate with antioxidant capacity.

The kinetics also differ: DPPH reached equilibrium after 120 min incubation (allowing slow-reacting compounds to contribute), while FRAP measures instant aneous reducing capacity after 30 min at 37 °C, potentially favoring different compound classes. Similar tissue-dependent antioxidant activity patterns have been reported in other Lamiaceae species [36].

## 4. Discussion

To the best of our knowledge, studies addressing UVB:PAR or UVA:PAR ratios, as well as plant responses to these ratios under horticultural growing conditions, are lacking or extremely scarce. Even studies that focus exclusively on the effects of UV radiation on the growth of horticultural crops are rare. UV radiation is associated with the accumulation of plant secondary metabolites (phenolic compounds, including flavonoids, etc.), which are related to plant color, taste, and perceived health-promoting attributes.

High solar radiation and elevated temperatures during the summer months can negatively affect both plant yield and quality. The incorporation of light-dispersive and reflective chromatic additives into photo-selective nets transforms direct sunlight into diffuse radiation, enabling deeper light penetration into the inner plant canopy. The radiometric properties of photo-selective nets are determined by their porosity and color. During cultivation, modifications in light quality beneath photo-selective shade nets can positively influence yield, quality traits, and phytochemical composition of aromatic and medicinal plants [37].

Colored shade nets alter light spectra, triggering biochemical changes in medicinal plants by manipulating photoreceptors (like phytochromes and cryptochromes) that regulate secondary metabolite (phenols, flavonoids, essential oils) production, photosynthesis, antioxidant activity, and pigmentation, often boosting beneficial compounds by shifting energy balance and influencing stress response pathways for enhanced medicinal quality [38].

Light regulates plant growth by controlling hormone balance (especially auxin), photosynthesis, and secondary metabolism; light intensity, quality, and shading are key factors, with shade promoting auxin synthesis and shoot elongation for shade avoidance. Light quantity is critical for *Lavandula* growth, flowering, essential oil (EO) content, and antioxidant production, with full sun (6–8+ h daily) being ideal for high-quality EOs and antioxidants in leaves and flowers. However, some studies suggest that shading with shade nets can increase the total yield of EO, fresh biomass, and dry mass, while decreasing non-fertile umbels, indicating a complex relationship where moderate shade can boost certain yields despite requiring a large amount of sun for optimal growth. The optimal balance between light and shade may depend on the specific *Lavandula* species and desired outcome.

Shading plants with photo-selective nets enhances the synthesis of essential oils compared to plants exposed to full sunlight [39]. Modifying light conditions could improve both the quantity and quality of essential oils in medicinal plants [7,8]. The intensity and quality of light can play an important role in the production of medicinal and aromatic plants and in the synthesis of essential oils. The optimal photo-selective net and shading level depends on the plant species and desired outcome, as each plant responds differently to light modification [7,8].

While this is well-studied in general, there is limited research on how light intensity specifically affects the growth, development, and secondary metabolism of lavender flowers and leaves. Shading can increase EO synthesis compared to full sun and can enhance biomass, leaf size, and branching depending on the specific light spectrum and intensity provided by the net.

Shading nets significantly alter plant morphology, often causing shade-avoidance responses like increased plant height, thinner stems, fewer branches, and reduced biomass (dry matter), while also delaying flowering, but the specific effects—like higher biomass/yield or altered flowering—depend heavily on the net’s color, shade intensity (e.g., 30–50% ideal), and the plant species’ sensitivity, as colored nets manipulate light spectrum (PAR, red/far-red) to control growth for better quality or yield in horticulture.

The content of lavender EO depended on both plant part and light conditions. The highest EO yield was recorded in flowers from plants grown under pearl shade nets and in leaves from plants grown under red nets The lowest EO content occurred in leaves and flowers from non-shaded (control) plants. The use of colored shade nets during the growth of different medicinal plants provides spectral changes, resulting in a higher content of EOs in sweet basil, mint, oregano, marjoram, thyme, dill [7,8], lemon balm [7], and sage [40]. EO content from different chemotypes of *Mentha longifolia* under shading is slightly higher than under control conditions [41].

The fact that essential oil (EO) content differs among individual plant parts, being higher in flowers (inflorescences) than in leaves and branches, is also confirmed by the results of several authors. EO yield in lavender leaves (0.50–0.99%), regardless of light intensity, in our study is comparable to the EO yields reported in neighboring countries. The content of essential oil was lower in leaves (0.21%) than in inflorescences (1.15%) [42]. There are data [43] about very low content (0.71–1.30%) in dry flowers.

In contrast, the essential oil yield in lavender flowers in our research (3.17–4.62%) is higher than the values reported in the literature for countries such as Poland [42], Bulgaria [44], Bosnia and Herzegovina [45], Croatia [46] and others.

Under the weather conditions in the study period, oil yields were within normal limits, compared with data from Bulgarian varieties of lavander(1.6–2.6%). This fact confirms that lavanderis adaptable to changes in the climate pattern [47]. Other sudies compared different lavender mcultivars at different geographic locations and practices and EO contentoscillated from 0.35% to 2% [48] and from 0.2 to [49].

The quality of EOs is influenced by environmental factors: climate and soil conditions, harvest time, post-harvest treatments, isolation methods, and variety origin—true lavenders (*L. angustifolia* Mill.) or indigenous lavender cultivar [50]. Different compositions were obtained in different experiments [51], which indicates the technological problems or concerns of different lavender subspecies [52].

The common criteria for determining *Lavandula* sp. essential oil quality are camphor, linalool, and linalyl acetate percentage [53]. The essential oil of *L. angustifolia* is highly valued due to the low content of camphor (up to 1.2% according to European Pharmacopoeia) and is much more expensive. Therefore, it is often mixed with cheaper oils of *L. latifolia* and *L. × intermedia* to achieve better quality that satisfies ISO 8902 standard [45]. Essential oil content and composition are primarily determined by plant genotype.

In our exploration, red nets yielded the highest number of identified LALEO components (65), while open field plants showed the lowest (55). The main compounds were 1,8-cineole, borneol, camphor, and linalool, with proportions depending on shading.

In our study, pearl nets resulted in the highest number of identified LAFEO components (59), while blue nets had the lowest (47). The dominant compound was 1,8-cineole, followed by linalool and borneol, with their proportions varying depending on shading treatment.

The composition of lavender essential oil (EO) differs between flowers and leaves and also depends on light modification by colored shading nets. 1,8-Cineole, an oxygenated monoterpene with a characteristic fresh, camphoraceous aroma, is the dominant EO component in both lavender flowers and leaves. Its lowest content was recorded in non-shaded (control) plants, while the highest was found in flowers of plants grown under red nets. The highest content in leaves was recorded in plants grown under blue nets.

Linalool is the second most abundant EO component. Its content in lavender flowers is significantly higher than in leaves, with the lowest value in non-shaded plants (24.7%) and the highest under pearl nets (27.3%). In leaves, linalool content is considerably lower, being lowest under red nets and slightly higher in non-shaded plants (8.6%).

Camphor content in the EO is markedly lower in flowers from plants grown under blue nets (2.5%) compared with non-shaded plants (8.6%). In LALEO, camphor content is significantly higher, almost twice that in LAFEO. Within LALEO, camphor content under blue nets (11.3%) is lower than in plants grown under pearl nets (13.9%).

The composition of the essential oils of lavender depended on the plant part and the stage of development too [48]. Similarly, as observed in our study, the main constituents of *L. angustifolia* cultivated in Belgrade were 1,8-cineole (7.1–48.4%), linalool (0.1–38.7%), borneol (10.9–27.7%), β-phellandrene (0.5–21.2%), and camphor (1.5–15.8%). In the shoots with flowers, inflorescences, and flowers, linalool is dominant; in the young leaves before flowering and old leaves, 1,8-cineole is dominant. In the young and incompletely developed leaves, β-phellandrene is dominant. Oxygen-containing monoterpenes predominated (88.9–91.3%), whereas all other compound groups were present in much lower amounts (Table 5).

The composition was compared with the parameters set out in the international standard for lavender oil. Similarly to our research, studies from neighboring countries also report a comparable distribution of the main components of lavender essential oil. Thus, the key constituents in the essential oil of *L. angustifolia* from Bulgaria were linalyl acetate, linalool, and β-caryophyllene [54], whereas in lavender from Italy, the following components are the most abundant: linalool, borneol, and 1,8-cineole 55]. The main components of the essential oil from *L. angustifolia* grown in Bosnia and Herzegovina were linalool and linalyl acetate, followed by α-pinene, lavandulol acetate, trans-caryophyllene, and others. In total, 24 components were identified [45].

The harvest time represents a key factor that strongly influences the chemical composition. Flowers collected in June have a higher content of phenolic compounds, while flowers harvested in September are a rich source of essential oils [55]. Sixteen compounds have been identified and monitored: the main one was linalyl acetate, followed by linalool, β-caryophyllene, cis-β-ocimene, lavandulyl acetateterpinen-4-ol, and trans-β ocimene (2.74–3.72%) [55].

Terpinen-4-ol is also an undesirable component that imparts a tart and moldy note to the oil [55]. It is also restricted in the international standard. In our study, content of terpinen-4-ol varies depending on the plant part and the color of the nets. Thus, the content in flowers ranges from 3.6% in flowers covered with red nets to 4.0% under pearl nets. Significantly lower contents are recorded in leaves, with lower values in non-shaded plants and under blue nets (2.0%) and slightly higher values under red nets (2.7%).

The oil from the inflorescences was dominated by linalool, caryophyllene oxide, and linalylacetate, while caryophyllene oxide, borneol, and geranyl acetate dominated in the leafy stems [45]. The essential oil obtained from the flowers has a milder fragrance than the one obtained from leaves or other plant parts. The essential oil from the leaves and stems is higher in 1,8-cineole and camphor, which are responsible for harsher notes. The linalyl acetate content determines the superior or inferior quality of lavender essential oil [56] and this constituent is also responsible for the floral–woody sensory character of the essential oil [57]. A camphor content exceeding 1.2% reduces the aroma quality by giving a fresher accent, while α-terpineol gives the desired lilac-like scent. It has been proposed that terpineol-4-ol in higher concentrations (over diminishes the essential oil value by giving it a grass-like scent [58]. The essential oil of the *Lavandula* from India contains less camphor (0.11%) and 1.8-cineole (1.14%) than the essential oil studied in our paper [59]. The major component was linalool (45.06%) followed by camphor (15.62%) and borneol (8.28%). Common criteria for the determination of oil quality are camphor, linalool, and linalyl acetate levels of essential oil [60]. According to the ISO 3515:2002 standard [61], lavender essential oil contains linalool (25–38%), linalyl acetate (25–45%), and camphor (0.5–1.0%), and lavandin essential oil contains linalool (24–35%), linalyl acetate (28–38%), and camphor (6–8%) according to the ISO 8902:2025 [62]. The characteristic scent of lavender oil is fresh floral, resembling the flowering tops of the plant (ISO 3515:2002). Oxygenated monoterpenes are the bulk constituent of lavender essential oil and responsible for the characteristic scent. The common monoterpenoids in lavender essential oil are alcohols, esters, ketones, and oxides (The content of trans-β ocimene meets the ISO 3515 [61] ). Gavric et al. [63] found that the cultivar greatly influences the antioxidative capacity. In addition to cultivars, environmental conditions such as temperature altitude, location, and agronomic practices can significantly impact antioxidant capacity. Lavander EO exhibited significant antioxidant activity, as measured by DPPH and FRAP assays. In our study, LAFEO samples from non-shaded (control) plants exhibited moderate antioxidant activity, showing the lowest EC50 values, representing the lowest activity among treatments, through still within the moderate range. Overall, flowers from non-shaded plants showed relatively higher radical scavenging activity compared to leaves across all shading treatments and incubation times. These EC50 value (20-50 mg/mL) classity the antioxidant activity as moderate according to established standards, which is appropriate for essential oils and relevant for cosmeticand pharmaceutical applications (Table 6).

The results are similar across other reported data. An antioxidant capacity of 80% ethanol flower extracts of *L. angustifolia* and lavandin ‘Budrovka’ grown in Croatia had an IC_50_ value of 10.62 µg mL^−1^ [48], while aqueous leaf and flower extracts of *L. angustifolia* grown in North Iran had an EC_50_ (50% scavenging concentration) value of 29.2 µg mL^−1^ [64]. A study by El Abdali et al. [65] exploring lavender antioxidant activity, as measured by DPPH and FRAP assays, obtained IC_50_ and EC_50_ values of 12.95 mg/mL and 11.88 mg/mL, respectively. The EO of lavender exhibited a total antioxidant capacity of 81.28 ± 2.28 mg AAE/g EO. Similarly, in Bosnia and Herzegovina, lavender EO obtained a higher antioxidant capacity—measured by FRAP assay (17.49 µM Fe^2+^/g)—than lavandin EO (10.84 µM Fe^2+^/g) [63]. Tested lavender EOs showed compliance with standard requirements for EO composition and exhibited moderate antioxidant capacity.

The contrasting results between DPPH and FRAP assays highlight the complexity of antioxidant assessment in essential oils. Our findings—where flowers excelled in DPPH but leaves in FRAP—align with reports in other aromatic plants showing tissue-specific antioxidant profiles [11]. The high linalool content in flowers (up to 27.3%) explains superior hydrogen-donating capacity in DPPH assays, as monoterpene alcohols are effective radical scavengers through hydrogen atom transfer [30].

Conversely, the elevated FRAP activity in leaves likely reflects (1) synergistic effects of camphor and borneol, whose combined electron-donating capacity exceeds individual contributions [32]; (2) the presence of unquantified phenolic compounds that accumulate preferentially in photosynthetic tissue and exhibit strong ferric-reducing activity [33]; and (3) potential matrix effects, as the more complex leaf essential oil composition (55–65 compounds vs. 47–59 in flowers) may create favorable conditions for electron transfer reactions. Importantly, these assays measure complementary rather than redundant aspects of antioxidant capacity. DPPH reflects biological relevance in lipid peroxidation scenarios (relevant for cosmetic applications), while FRAP indicates potential in metal chelation and redox regulation (relevant for pharmaceutical use).

Further research aimed at optimizing lavender agrotechnology should focus on creating favorable microclimatic conditions and modifying light through shading in order to achieve higher yields and superior essential oil quality. Nevertheless, this preliminary study evaluating both the yield and quality of lavender essential oil under colored shading nets highlights the strong potential of this species for expanded production in southern Serbia.

## 5. Conclusions

Based on the results, shading lavender with pearl and red nets increased essential oil (EO) yield in both plant parts compared to non-shaded plants. The most abundant EO component was 1,8-cineole (eucalyptol); the highest content in flower essential oil (LAFEO, 32.2%) was obtained from plants covered with red nets, whereas the highest content in leaf essential oil (LALEO, 39.8%) was recorded in plants shaded with blue nets. Shading with colored nets differentially affected the content of individual essential oil components. A higher camphor content in flowers, which negatively affects oil quality, was observed in non-shaded plants. However, essential oil from non-shaded plants indicated stronger antioxidant capacity, compared to all shading treatments. Nevertheless, this preliminary study, which evaluated both the yield and quality of lavender essential oil under colored shading nets, highlights the strong potential of this species for expanded production in southern Serbia.

## Figures and Tables

**Table 1:** Average value of PPFD (µmol/m^2^/s) and solar radiation (W/m^2^) in May, June, July, and August 2024.

Month	PPFD				W/m^2^			
	Control—Non-Shade	Pearl	Red	Blue	Control—Non-Shade	Pearl	Red	Blue
May	1530.3	793.6	814.2	770.1	674.7	389.1	403.9	331.7
June	1928.0	978.6	1004.1	946.5	866.2	564.1	599.3	514.8
July	2025.8	1105.4	1193.9	1018.3	996.0	603.6	647.2	563.8
August	1867.4	961.7	993.6	937.2	789.1	483.8	501.1	442.0

**Table 2:** Effect of shading by color shade nets on morphological characterics and yield parameters of *L. angustifolia*.

Shade Nets	Plant Height (cm)	Number of Primary Branches	Total Number of Branches	Vegetative Biomass g/Plant	Vegetative Biomass t/ha	Fresh Inflorescence Mass (g/Plant)	Fresh Inflorescence Mass g/m^2^	Inflorescence Length (cm)	Dry Inflorescence Mass (g/Plant)	Dry Inflorescence Mass g/m^2^	Fresh-to-Dry Inflorescence Mass Ratio
Pearl	28.6 a a	3.24 a a	16.4 a a	284.4 a a	7.90 a a	47.3 a a	132.40 a a	6.7 a a	26.740 a a	74.76 a a	1.77
Red	27.9 ab a	3.21 a a	15.6 a a	255.3 ab ab	7.11 ab ab	46.52 a a	130.28 a a	6.4 ab a	27.023 a a	75.65 a a	1.72
Blue	24.2 bc ab	2.98 ab a	14.1 b ab	218.4 bc ab	6.10 bc b	25.79 b b	72.197 b b	5.9 ab a	14.327 b b	40.08 b b	1.80
Control	22.1 c b	2.73 b a	13.7 b b	204.6 c b	5.69 c b	27.92 b b	78.189 b b	5.5 b a	15.393 b b	43.09 b b	1.81

Values followed by different letters are significantly different at *p* < 0.05 and *p* < 0.01.

**Table 3:** Effect of shading on lavender essential oil yield (LAEO).

Shade Nets	Mean ± SD (mL/100 g p.m.)	
	Flowers (LAFEO)	Leaves (LALEO)
Pearl	4.62 ± 0.03 a	0.59 ± 0.02 b
Red	4.39 ± 0.09 a	0.99 ± 0.07 a
Blue	3.71 ± 0.13 b	0.65 ± 0.02 b
Control	3.17 ± 0.13 c	0.50 ± 0.03 b

Values followed by different letters are significantly different at *p* < 0.05.

**Table 4:** Chemical composition of lavender flower essential oil (LAFEO).

No.	*t*_ret_ min	Compound	RI ^exp^	RI ^lit^	Method of Identification	Content %			
						Pearl	Red	Blue	Control
1.	6.28	Tricyclene	916	921 ^a^ [28]	RI, MS	tr	tr	tr	tr
2.	6.38	α-Thujene	920	924 ^a^	RI, MS	tr	tr	tr	tr
3.	6.58	α-Pinene	927	932 ^a^	RI, MS	0.2	0.4	0.2	0.2
4.	7.02	Camphene	943	946 ^a^	RI, MS	0.2	0.4	0.2	0.2
5.	7.17	Thuja-2,4(10)-diene	949	953 ^a^	RI, MS	-	tr	tr	tr
6.	7.67	Verbenene	967	961 ^a^	RI, MS	-	tr	tr	tr
7.	7.74	Sabinene	969	969 ^a^	RI, MS	0.1	0.2	0.1	0.1
8.	7.86	β-Pinene	973	974 ^a^	RI, MS	0.3	0.7	0.4	0.4
9.	8.02	1-Octen-3-ol	979	974 ^a^	RI, MS	0.5	0.4	0.4	0.4
10.	8.12	3-Octanone	983	979 ^a^	RI, MS	0.3	0.2	0.2	0.2
11.	8.25	Myrcene	988	988 ^a^	RI, MS	tr	0.1	tr	tr
12.	8.27	dehydro-1,8-Cineole	988	988 ^a^	RI, MS	tr	tr	tr	tr
13.	8.37	Butyl butanoate	992	993 ^a^	RI, MS	tr	tr	tr	tr
14.	8.92	δ-3-Carene	1009	1008 ^a^	RI, MS	tr	tr	-	0.5
15.	8.97	Hexyl acetate	1010	1007 ^a^	RI, MS	0.6	0.6	0.6	tr
16.	9.15	α-Terpinene	1016	1014 ^a^	RI, MS	tr	tr	tr	tr
17.	9.37	*p*-Cymene	1021	1020 ^a^	RI, MS	tr	tr	tr	0.3
18.	9.48	o-Cymene	1024	1022 ^a^	RI, MS	0.3	0.6	0.3	tr
19.	9.73	1,8-Cineole	1031	1026 ^a^	RI, MS	29.2	32.2	30.7	27.4
20.	10.26	(*E*)-β-Ocimene	1046	1044 ^a^	RI, MS	-	tr	tr	tr
21.	10.67	γ-Terpinene	1057	1054 ^a^	RI, MS, Co-I	tr	0.2	0.2	tr
22.	11.15	*cis*-Sabinene hydrate	1070	1065	RI, MS	0.7	0.2	0.4	0.1
23.	11.28	*cis*-Linalool oxide (furanoid)	1074	1067 ^a^	RI, MS	3.1	2.0	2.7	0.7
24.	11.69	Camphenilone	1085	1078 ^a^	RI, MS	tr	tr	-	2.2
25.	11.88	*trans*-Linalool oxide (furanoid)	1090	1084 ^a^	RI, MS	2.1	1.3	1.8	1.4
26.	11.93	*p*-Cymenene	1088	1089 ^a^	RI, MS	-	-	-	tr
27.	12.16	6-Camphenone	1094	1095 ^a^	RI, MS	-	-	-	tr
28.	12.52	Linalool	1105	1095 ^a^	RI, MS, Co-I	27.3	24.7	25.9	24.7
29.	12.98	*cis*-*p*-Menth-2-en-1-ol	1119	1118 ^a^	RI, MS	-	0.1	-	tr
30.	13.32	dehydro-Sabina ketone	1127	1117 ^a^	RI, MS	tr	tr	-	tr
31.	13.38	α-Campholenal	1124	1122 ^a^	RI, MS	-	-	-	tr
32.	13.39	α-Campholenal	1128	1122 ^a^	RI, MS	tr	tr	-	tr
33.	13.87	Nopinone	1140	1135 ^a^	RI, MS	tr	tr	-	tr
34.	14.05	*trans*-Sabinol	1145	1137 ^a^	RI, MS	tr	tr	-	
35.	14.14	Camphor	1147	1141 ^a^	RI, MS	7.9	8.0	7.5	8.6
36.	14.30	2-(1*Z*)-propenyl-Phenol	1146	1146 ^a^	RI, MS	-	-	tr	tr
37.	14.87	Pinocarvone	1164	1160	RI, MS	0.2	0.3	0.2	0.3
38.	15.29	Borneol	1175	1165 ^a^	RI, MS	18.0	18.2	18.5	21.9
39.	15.64	Terpinen-4-ol	1183	1174 ^a^	RI, MS	4.0	3.6	3.7	3.7
40.	16.02	Hexyl butanoate	1192	1191 ^a^	RI, MS	0.9	1.6	1.2	1.2
41.	16.14	*p*-Cymen-8-ol	1190	1185 ^b^ [29]	RI, MS	tr	tr	-	0.2
42.	16.26	α-Terpineol	1196	1186 ^a^	RI, MS	0.9	1.2	1.0	1.2
43.	16.47	Myrtenol	1203	1194 ^a^	RI, MS	-	tr	-	tr
44.	16.88	Verbenone	1213	1204 ^a^	RI, MS	tr	tr	tr	0.2
45.	17.57	Isobornyl formate	1230	1235 ^a^	RI, MS	0.6	0.7	0.7	0.7
46.	17.88	Hexyl 2-methylbutanoate	1237	1227 ^c^ [30]	RI, MS	0.2	0.4	0.2	0.2
47.	18.09	Hexyl isovalerate	1242	1241 ^a^	RI, MS	tr	tr	tr	tr
48.	18.24	Cumin aldehyde	1245	1238 ^a^	RI, MS	-	tr	tr	tr
49.	18.36	Carvone	1248	1239 ^a^	RI, MS, Co-I	tr	0.2	tr	0.2
50.	18.70	Linalool acetate	1256	1254 ^a^	RI, MS, Co-I	1.1	0.3	0.9	1.3
51.	18.85	*trans*-Sabinene hydrate acetate	1260	1253 ^a^	RI, MS	-	tr	-	
52.	19.98	Isobornyl acetate	1286	1283 ^a^	RI, MS	tr	tr	-	
53.	20.18	Lavandulyl acetate	1291	1288 ^a^	RI, MS	0.2	0.4	0.3	0.3
54.	21.90	Hexyl tiglate	1332	1330 ^a^	RI, MS	tr	tr	tr	tr
55.	23.28	Linalool isobutanoate	1366	1373 ^a^	RI, MS	tr	tr	-	-
56.	24.06	Geranyl acetate	1384	1379 ^a^	RI, MS	-	0.1	-	-
57.	24.08	Linalool isobutanoate	1378	1373 ^a^	RI, MS	-	-	-	tr
58.	24.14	Hexyl hexanoate	1387	1382 ^a^	RI, MS	0.2	0.2	0.2	tr
59.	24.44	β-Elemene	1393	1389 ^a^	RI, MS	tr	-	-	-
60.	25.53	(*E*)-Caryophyllene	1420	1417 ^a^	RI, MS	tr	-	-	-
61.	26.92	α-Humulene	1455	1452 ^a^	RI, MS	tr	-	-	-
62.	27.83	γ-Muurolene	1478	1478 ^a^	RI, MS	tr	-	-	-
63.	28.26	β-Selinene	1488	1489 ^a^	RI, MS	tr	-	-	-
64.	28.77	α-Muurolene	1501	1500 ^a^	RI, MS	tr	-	-	-
65.	29.08	Lavandulyl isovalerate	1509	1509 ^a^	RI, MS	-	tr	tr	tr
66.	29.33	γ-Cadinene	1516	1513 ^a^	RI, MS	tr	tr	0.2	tr
67.	29.66	δ-Cadinene	1525	1522 ^a^	RI, MS	tr	-	-	-
68.	31.94	β-Copaen-4-α-ol	1584	1590 ^a^	RI, MS	tr	-	-	-
69.	32.01	Caryophyllene oxide	1586	1582 ^a^	RI, MS	0.8	0.7	0.8	0.8
70.	33.02	2,(7Z)-Bisaboladien-4-ol	1613	1618 ^a^	RI, MS	0.1	-	-	-
71.	33.75	Muurola-4,10(14)-dien-1-β-ol	1633	1630 ^a^	RI, MS	tr	-	-	-
72.	34.33	epi-α-Cadinol	1639	1638 ^a^	RI, MS	-	-	0.3	0.4
73.	34.80	7-epi-α-Eudesmol	1661	1662 ^a^	RI, MS	tr	-	-	-
	Total number of constituents					59	57	47	57
	Total identified (%)					100.0	100.0	100.0	100.0
	Grouped components (%)								
	Monoterpene hydrocarbons (1–6, 9, 12, 14–16, 18)					1.1	1.4	1.4	1.3
	Oxygen-containing monoterpenes (10, 17, 19–31, 33–36, 39–42, 44)					95.3	94.5	94.5	95.0
	Sesquiterpene hydrocarbons (46–53)					tr	0.2	0.2	tr
	Oxygen-containing sesquiterpenes (54–59)					0.9	1.1	1.1	1.2
	Others (7–11, 13, 15, 32, 34–38, 42–47, 49–51, 53, 54)					2.7	2.8	2.8	2.5

*t*_ret_: retention time; RI ^lit^–retention indices from the literature; RI ^exp^: experimentally determined retention indices using a homologous series of *n*-alkanes (C_8_-C_20_) on the HP-5MS column. MS: constituent identified by mass-spectra comparison; RI: constituent identified by retention index matching; Co-I: constituent identity confirmed by GC co-injection of an authentic sample; tr = trace amount (<0.05%). RI ^lit^ (the retention index) was determined according to the different references (a, b, c).

**Table 5:** Chemical composition of lavender leaves essential oil (LALEO).

No.	*t*_ret_ min	Compound	RI ^exp^	RI ^lit^	Method of Identification	Content %			
						Pearl	Red	Blue	Control
1.	6.28	Tricyclene	916	921 ^a^ [28]	RI, MS	tr	tr	tr	tr
2.	6.38	α-Thujene	920	924 ^a^	RI, MS	tr	tr	tr	tr
3.	6.58	α-Pinene	927	932 ^a^	RI, MS	0.4	0.3	0.8	0.6
4.	7.02	Camphene	943	946 ^a^	RI, MS	0.3	0.3	0.6	0.5
5.	7.17	Thuja-2,4(10)-diene	949	953 ^a^	RI, MS	-	tr	tr	tr
6.	7.67	Verbenene	967	961 ^a^	RI, MS	tr	tr	0.1	tr
7.	7.74	Sabinene	969	969 ^a^	RI, MS	0.2	0.1	0.2	0.2
8.	7.86	β-Pinene	973	974 ^a^	RI, MS	0.8	0.6	1.3	1.1
9.	8.02	1-Octen-3-ol	979	974 ^a^	RI, MS	0.2	0.2	0.2	0.1
10.	8.12	3-Octanone	983	979 ^a^	RI, MS	tr	tr	tr	tr
11.	8.25	Myrcene	988	988 ^a^	RI, MS	tr	tr	0.1	0.2
12.	8.27	dehydro-1,8-Cineole	988	988 ^a^	RI, MS	tr	0.1	tr	tr
13.	8.37	Butyl butanoate	992	993 ^a^	RI, MS	tr	tr	tr	tr
14.	8.92	δ-3-Carene	1009	1008 ^a^	RI, MS	0.2	0.1	0.2	0.1
15.	8.97	Hexyl acetate	1010	1007 ^a^	RI, MS	tr	0.1	tr	0.1
16.	9.15	α-Terpinene	1016	1014 ^a^	RI, MS	tr	tr	0.3	tr
17.	9.37	*p*-Cymene	1021	1020 ^a^	RI, MS	0.3	0.2	1.1	0.3
18.	9.48	o-Cymene	1024	1022 ^a^	RI, MS	0.9	0.8	tr	1.0
19.	9.73	1,8-Cineole	1031	1026 ^a^	RI, MS	33.9	30.4	39.8	34.3
20.	9.86	(*Z*)-β-Ocimene	1033	1032 ^a^	RI, MS	-	-	tr	0.8
21.	10.26	(*E*)-β-Ocimene	1044	1044 ^a^	RI, MS	-	-	tr	tr
22.	10.67	γ-Terpinene	1057	1054 ^a^	RI, MS, Co-I	0.1	0.1	0.2	0.1
23.	11.15	*cis*-Sabinene hydrate	1070	1065		0.1	0.3	0.3	0.4
24.	11.28	*cis*-Linalool oxide (furanoid)	1074	1067 ^a^	RI, MS	0.3	0.9	0.4	0.3
25.	11.66	*meta*-Cymenene	1081	1082 ^a^	RI, MS	-	-	tr	-
26.	11.69	Camphenilone	1085	1078 ^a^	RI, MS	tr	tr	-	-
27.	11.82	Terpinolene	1085	1086 ^a^	RI, MS	-	-	tr	tr
28.	11.88	*trans*-Linalool oxide (furanoid)	1090	1084 ^a^	RI, MS	0.4	0.7	0.5	0.2
29.	12.13	6-Camphenone	1098	1095	RI, MS	tr	tr	tr	-
30.	12.52	Linalool	1105	1095 ^a^	RI, MS, Co-I	6.5	8.0	6.0	8.6
31.	13.32	dehydro-Sabina ketone	1127	1117 ^a^	RI, MS	0.1	tr	0.1	0.1
32.	13.39	α-Campholenal	1128	1122 ^a^	RI, MS	0.3	0.1	0.2	0.2
33.	13.87	Nopinone	1140	1135 ^a^	RI, MS	0.2	0.2	0.2	0.1
34.	14.05	*trans*-Sabinol	1145	1137 ^a^	RI, MS	tr	0.2	-	**-**
35.	14.14	Camphor	1147	1141 ^a^	RI, MS	13.9	12.6	11.3	12.4
36.	14.87	Pinocarvone	1164	1160 ^a^	RI, MS	0.9	0.8	0.2	0.6
37.	14.30	2-(1*Z*)-propenyl-Phenol	1146	1146 ^a^	RI, MS	-	-	tr	tr
38.	15.29	Borneol	1175	1165 ^a^	RI, MS	26.5	26.3	21.9	24.5
39.	15.64	Terpinen-4-ol	1183	1174 ^a^	RI, MS	2.5	2.7	2.0	2.0
40.	16.02	Hexyl butanoate	1192	1191 ^a^	RI, MS	2.1	2.0	2.3	2.2
41.	16.14	*p*-Cymen-8-ol	1190	1185 ^b^ [29]	RI, MS	0.4	0.5	0.4	0.2
42.	16.26	α-Terpineol	1196	1186 ^a^	RI, MS	1.7	1.9	1.6	1.2
43.	16.46	Myrtenol	1204	1194 ^a^	RI, MS	0.5	0.7	0.4	tr
44.	16.88	Verbenone	1213	1204 ^a^	RI, MS	0.3	0.4	0.2	0.2
45.	17.38	(Z)-Ocimenone	1225	1226 ^a^	RI, MS	-	tr	-	-
46.	17.57	Isobornyl formate	1230	1235 ^a^	RI, MS	1.1	1.2	1.0	1.1
47.	17.88	Hexyl 2-methylbutanoate	1237	1227 ^c^ [30]	RI, MS	0.4	0.4	0.6	0.6
48.	18.09	Hexyl isovalerate	1242	1241 ^a^	RI, MS	tr	0.1	0.1	0.1
49.	18.22	Cumin aldehyde	1245	1238 ^a^	RI, MS	0.3	0.3	0.2	0.2
50.	18.36	Carvone	1248	1239 ^a^	RI, MS, Co-I	0.4	0.5	0.4	0.4
51.	18.70	Linalool acetate	1256	1254 ^a^	RI, MS, Co-I	0.5	0.3	0.7	1.0
52.	18.85	*trans*-Sabinene hydrate acetate	1260	1253 ^a^	RI, MS	-	0.2	-	-
53.	19.98	Isobornyl acetate	1286	1283 ^a^	RI, MS	0.1	0.2	0.1	0.1
54.	20.18	Lavandulyl acetate	1291	1288 ^a^	RI, MS	0.3	0.3	0.4	0.4
55.	20.67	*p*-Cymen-7-ol	1303	1290 ^c^	RI, MS	-	tr	-	
56.	21.48	Carvacrol	1322	1312 ^d^ [31]	RI, MS	-	tr	-	
57.	21.90	Hexyl tiglate	1332	1330 ^a^	RI, MS	0.1	0.2	0.2	0.2
58.	23.28	Linalool isobutanoate	1366	1373 ^a^	RI, MS	tr	0.1	-	0.2
59.	24.06	Geranyl acetate	1385	1379 ^a^	RI, MS	tr	tr	-	-
60.	24.14	Hexyl hexanoate	1387	1382 ^a^	RI, MS	0.5	0.6	0.7	0.4
61.	24.44	β-Elemene	1393	1389 ^a^	RI, MS	tr	-	-	-
62.	25.53	(*E*)-Caryophyllene	1420	1417 ^a^	RI, MS	tr	-	-	-
63.	26.92	α-Humulene	1455	1452 ^a^	RI, MS	tr	-	-	-
64.	26.99	(*E*)-β-Farnesene	1457	1454 ^a^	RI, MS	-	0.1	-	-
65.	27.31	Linalool isovalerate	1465	1466 ^a^	RI, MS	-	tr	-	-
66.	29.08	Lavandulyl isovalerate	1509	1509 ^a^	RI, MS	0.2	0.3	0.2	0.2
67.	29.33	γ-Cadinene	1516	1513 ^a^	RI, MS	0.2	0.3	0.2	0.2
68.	29.66	δ-Cadinene	1525	1522 ^a^	RI, MS	tr	-	-	-
69.	30.82	epi-Longipinanol	1555	1562 ^a^	RI, MS	-	0.1	-	-
70.	32.01	Caryophyllene oxide	1586	1582 ^a^	RI, MS	1.5	2.1	1.5	1.6
71.	33.02	2,(7 Z)-Bisaboladien-4-ol	1613	1618 ^a^	RI, MS	tr	-	-	-
72.	33.26	1,10-di-epi-Cubenol	1619	1618 ^a^	RI, MS	-	0.1	-	-
73.	34.33	epi-α-Cadinol	1649	1644 ^a^	RI, MS	-	0.9	0.4	0.5
74.	35.49	Khusinol	1681	1679 ^a^	RI, MS	-	0.1	-	-
75.	35.81	α-Bisabolol	1690	1685 ^a^	RI, MS	-	tr	-	-
	Number of constituent					60	65	58	55
	Total identified (%)					100.0	100.0	100.0	100.0
	Grouped components (%)								
	Monoterpene hydrocarbons (1–7, 10, 13, 15–17, 19)					3.2	2.5	4.9	4.9
	Oxygen-containing monoterpenes (11, 18, 20–33, 35–39, 42–46, 48, 49, 54)					91.3	90.2	88.9	89.1
	Sesquiterpene hydrocarbons (51–53, 55, 56)					0.2	0.5	0.2	0.2
	Oxygen-containing sesquiterpenes (57–60)					2.0	3.2	1.9	2.1
	Others (8–15, 32, 34–36, 38, 40–45, 47, 49–53)					3.3	3.6	4.1	3.7

*t*_ret_: retention time; RI ^lit^–retention indices from the literature; RI ^exp^: experimentally determined retention indices using a homologous series of *n*-alkanes (C_8_-C_20_) on the HP-5MS column. MS: constituent identified by mass-spectra comparison; RI: constituent identified by retention index matching; Co-I: constituent identity confirmed by GC co-injection of an authentic sample; tr = trace amount (<0.05%). RI ^lit^ (the retention index) was determined according to the different references (a, b, c, d) [28,29,30,31].

**Table 6:** DPPH radical neutralization activity of lavender essential oil after 20, 60, and 120 min incubation.

Shade Plant		EC_50_ (mg/mL)		
Nets Organ		20 min Incubation	60 min Incubation	120 min Incubation
Pearl	flowers	78.577 ± 0.734	49.115 ± 0.483	35.308 ± 0.156
	leaves	78.368 ± 3.066	44.595 ± 0.424	42.397 ± 0.300
Red	flowers	54.019 ± 1.023	40.361 ± 0.519	33.098 ± 0.163
	leaves	55.159 ± 1.368	37.695 ± 0.153	27.139 ± 0.199
Blue	flowers	56.918 ± 0.366	35.721 ± 0.385	26.526 ± 0.242
	leaves	69.130 ± 1.029	48.271 ± 0.948	28.610 ± 0.234
Control	flowers	51.972 ± 0.279	32.255 ± 0.165	20.263 ± 0.105
	leaves	71.610 ± 1.141	34.247 ± 0.321	24.335 ± 0.133

**Table 7:** FRAP values of essential oils obtained from flowers and leaves.

Shade Nets	mg EFe^2+^/g Essential Oil (Mean ± SD)	
	Flowers	Leaves
Pearl	0.33 ± 0.004 c	0.42 ± 0.004 b
Red	0.28 ± 0.004 c	0.72 ± 0.004 a
Blue	0.46 ± 0.004 b	0.51 ± 0.004 b
Control	0.59 ± 0.004 a	0.68 ± 0.004 a

Values followed by different letters are significantly different at *p* < 0.05.

## Data Availability

All the data is available in the manuscript file.

## References

[1] Wu B.S., Mansoori M., Schwalb M., Islam S., Naznin M.T., Addo P.W., MacPherson S., Orsat V., Lefsrud M. (2024). Light emitting diode effect of red, blue, and amber light on photosynthesis and plant growth parameters. J. Photochem. Photobiol. B Biol..

[2] Wang J., Yao R., Sun Z., Wang M., Jiang C., Zhao X., Liu X., Zhong C., Zhang H., Zhao S. (2024). Effects of shading on morphology, photosynthesis characteristics, and yield of different shade-tolerant peanut varieties at the flowering stage. Front. Plant Sci..

[3] Wei Y., Wang S., Yu D. (2023). The role of light quality in regulating early seedling development. Plants.

[4] Arthurs S.P., Stamps R.H., Giglia F.F. (2013). Environmental modification inside photoselective shade houses. Hortic. Sci..

[5] Ilić S.Z., Fallik E. (2017). Light quality manipulation improves vegetables quality at harvest and postharvest: A review. Environ. Exp. Bot..

[6] Oliveira G.C., Vieira W.L., Bertolli S.C., Pacheco A.C. (2016). Photosynthetic behavior, growth and essential oil production of Melissa officinalis L. cultivated under colored shade nets. Chil. J. Agric. Res..

[7] Ilić S.Z., Milenković L., Tmušić N., Stanojević L.J., Cvetković D. (2022). Essential oils content, composition and antioxidant activity of lemon balm, mint and sweet basil from Serbia. LWT Food Sci. Technol..

[8] Milenković L., Ilić Z.S., Stanojević L., Danilović B., Šunić L., Kevrešan Ž., Stanojević J., Cvetković D. (2024). Chemical composition and bioactivity of dill seed (*Anethum graveolens* L.) essential oil from plants grown under shading. Plants.

[9] Hubert-Schöler C., Tsiaparas S., Luhmer K., Moll M.D., Passon M., Wüst M., Schieber A., Pude R. (2025). Quality and physiology of selected mentha genotypes under coloured shading nets. Agronomy.

[10] Şeker S., Çakaloğulları U., Bayram E., Tatar O. (2023). Production of sage, oregano and rosemary under shading conditions and the effects of light on growth and essential oil properties. Ind. Crops Prod..

[11] Milenković L., Ilić Z.S., Stanojević L., Šunić L., Milenković A., Stanojević J., Cvetković D. (2025). Does photoselective netting influence yield, chemical composition and antioxidant activities of essential oils in cultivated sage?. Front. Plant Sci..

[12] Najafian S., Afshar M., Radi M. (2022). Annual phytochemical variations and antioxidant activity within the aerial parts of *Lavandula angustifolia*, an evergreen medicinal plant. Chem. Biodiv.

[13] Mijatovic S., Stankovic J.A., Calovski I.C., Dubljanin E., Pljevljakusic D., Bigovic D., Dzamic A. (2022). Antifungal activity of *Lavandula angustifolia* essential oil against *Candida albicans*: Time-kill study on pediatric sputum isolates. Molecules.

[14] Dobros N., Zawada K., Paradowska K. (2022). Phytochemical profile and antioxidant activity of *Lavandula angustifolia* and *Lavandula intermedia* cultivars extracted with different methods. Antioxidants.

[15] Stanojević L.J., Stanković M., Cakić M., Nikolić V., Nikolić L.J., Ilić D., Radulović N. (2011). The effect of hydrodisillation techniques on yield, kinetics, composition and antimicrobial activity of essential oils from flowers of *Lavandula officinalis* L.. Hem. Ind..

[16] Aprotosoaie A.C., Gille E., Trifan A., Luca V.S., Miron A. (2017). Essential oils of Lavandula genus: A systematic review of their chemistry. Phytochem. Rev..

[17] Crișan I., Ona A., Vârban D., Muntean L., Vârban R., Stoie A., Mihăiescu T., Morea A. (2023). Current trends for lavender (*Lavandula angustifolia* Mill.) crops and products with emphasis on essential oil quality. Plants.

[18] Aćimović M., Lončar B., Stanković-Jeremić J., Cvetković M., Pezo L., Pezo M., Todosijević M., Tešević V. (2022). Weather conditions influence on lavandin essential oil and hydrolate quality. Horticulturae.

[19] Council of Europe (2006). European Pharmacopoeia.

[20] Perović A., Karabegović I., Krstić M., Veličković A., Avramović J., Danilović B., Đorđević N., Mančić S., Veljković V. (2025). Optimization and kinetics of lavender essential oil extraction: Effects of flower pretreatment, salt solutions, and hydrolat reuse. Adv. Techol..

[21] Nurzyńska-Wierdak R., Zawiślak G. (2016). Chemical composition and antioxidantactivity of lavender (*Lavandula angustifolia* Mill.) above ground parts. Acta Sci. Pol. Hort. Cultus.

[22] Busarčević V., Stupar D. (2000). Jugoslovenska Farmakopeja, Ph. Jug. V.

[23] Stanojević J.S., Stanojević L.P., Cvetković D.J., Danilović B.R. (2015). Chemical composition, antioxidant and antimicrobial activity of the turmeric essential oil (*Curcuma longa* L.). Adv. technol..

[24] Benzie I.F., Strain J.J. (1996). The ferric reducing ability of plasma (FRAP) as a measure of “antioxidant power”: The FRAP assay. Anal. Biochem..

[25] Stanojević L.P., Zdravković A.S., Stanković M.Z., Cakić M.D., Nikolić V.D., Ilić D.P. (2013). The antioxidant activity of aqueous ethanolic extracts from nettle leaf (Urtica dioica L.). Savrem. Tehnol..

[26] Milenković A., Aleksovski S., Miteva K., Milenković L., Stanojević J., Nikolić G., Ilić Z.S., Stanojević L. (2025). The effect of extraction technique on the yield, extraction kinetics and antioxidant activity of black pepper (*Piper nigrum* L.) ethanolic extracts. Horticulturae.

[27] Zhang Y., Wang D., Li H., Bai H., Sun M., Shi L. (2023). Formation mechanism of glandular trichomes involved in the synthesis and storage of terpenoids in lavender. BMC Plant Biol..

[28] Adams R.P. (2007). Identification of Essential Oil Components by Gas Chromatography/Mass Spectrometry.

[29] Benkaci-Ali F., Baaliouamer A., Meklati B.Y., Chemat F. (2007). Chemical composition of seed essential oils from Algerian *Nigella sativa* extracted by microwave and hydrodistillation. Flavour. Fragr. J..

[30] Saroglou V., Dorizas N., Kypriotakis Z., Skaltsa H.D. (2006). Analysis of the essential oil composition of eight Anthemis species from Greece. J. Chromatogr. A.

[31] Zouari N., Ayadi I., Fakhfakh N., Rebai A., Zouari S. (2012). Variations of chemical composition of essential oils in wild-populations of *Thymus algeriensis* Boiss et Reut., a North African endemic species. Lipids Health Dis..

[32] Molyneux P. (2004). The use of the stable free radical diphenylpicrylhydrazyl (DPPH) for estimating antioxidant activity. Songklanakarin J. Sci. Technol..

[33] Gulcin I., Alwasel S.H. (2023). DPPH Radical Scavenging Assay. Processes.

[34] Baliyan S., Mukherjee R., Priyadarshini A., Vibhuti A., Gupta A., Pandey R.P., Chang C.M. (2022). Determination of antioxidants by DPPH radical scavenging activity and quantitative phytochemical analysis of *Ficus religiosa*. Molecules.

[35] Proestos C., Komaitis M. (2013). Analysis of Naturally Occurring Phenolic Compounds in Aromatic Plants by RP-HPLC Coupled to Diode Array Detector (DAD) and GC-MS after Silylation. Foods.

[36] Macedo Arantes S., Teresa Caldeira A., Rosário Martins M. (2022). Essential Oils High in 1,8-Cineole of Mediterranean flavoring Plants: Health Benefits.

[37] Kotilainen T., Robson T.M., Hernandez R. (2018). Light quality characterization under climate screens and shade nets for controlled-environment agriculture. PLoS ONE.

[38] Tmušić N., Ilić Z.S., Milenković L., Šunić L., Lalević D., Kevrešan Ž., Mastilović J., Stanojević L., Cvetković D. (2021). Shading of Medical Plants Affects the Phytochemical Quality of Herbal Extracts. Horticulturae.

[39] Ilić S.Z., Milenković L., Šunić L., Tmušić N., Mastilović J., Kevrešan Ž., Stanojević L., Danilović B., Stanojević J. (2021). Efficiency of basil essential oil antimicrobial agents under different shading treatments and harvest times. Agronomy.

[40] Li Y., Craker L.E., Potter T. (1996). Effect of light level on the essential oil production of sage (*Salvia officinalis*) and thyme (*Thymus vulgaris*). Acta Hortic..

[41] Hubert-Schöler C., Tsiaparas S., Luhmer K., Moll M.D., Passon M., Wüst M., Schieber A., Pude R. (2024). Essential oil composition and physiology of three *Mentha* genotypes under shaded field conditions. Plants.

[42] Zawadzinska A., Wesołowska A., Skutnik E., Rabiza-Swider J., Salachna P. (2025). Changes in growth and chemical composition of the essential oil from flowers and leafy stems of *Lavandula angustifolia* grown in media amended with bark and sewage sludge. Molecules.

[43] Zheljazkov V., Astatkie T., Hristov A. (2012). Lavender and hyssop productivity, oil content, and bioactivity as function of harvest time and drying. Ind. Crops Prod..

[44] Konakchiev A. (2015). Essential Oils of *Lavandula angustifolia* Mill. Varieties and *Achillea* L. Species. Ph.D. Thesis.

[45] Talić S., Odak I., Marković Boras M., Smoljan A., Martinović Bevanda A. (2023). Essential oil and extracts from *Lavandula angustifolia* Mill. cultivated in Bosnia and Herzegovina: Antioxidant activity and acetylcholinesterase inhibition. Int. J. Plant Based Pharm..

[46] Blažeković B., Vladimir-Knežević S., Brantner A., Štefan M.B. (2010). Evaluation of antioxidant potential of *Lavandula x intermedia* Emeric ex Loisel. ‘Budrovka’: A comparative study with *L. angustifolia* Mill. Molecules.

[47] Kara N., Bayda H. (2013). Determination of lavender and lavandin cultivars (*Lavandula* sp.) containing high quality essential oil in Isparta, Turkey. Turk. J. Field Crops.

[48] Lakušić B., Lakušić D., Ristić M., Marčetić M., Slavkovska V. (2014). Seasonal Variations in the Composition of the Essential Oils of *Lavandula angustifolia* (Lamiacae). Nat. Prod. Comm..

[49] Kiprovski B., Zeremski T., Varga A., Cabarkapa I., Filipović J., Lončar B., Aćimović M. (2023). Essential oil quality of lavender grown outside its native distribution range: A study from Serbia. Horticulturae.

[50] Chrysargyris A., Panayiotou C., Tzortzakis N. (2016). Nitrogen and phosphorus levels affected plant growth, essential oil composition and antioxidant status of lavender plant (*Lavandula angustifolia* Mill.). Ind. Crops Prod..

[51] Silva S., Lus J., Nogueira P., Blank A., Sampaio T., Pinto J., Junior A. (2017). Organo-mineral fertilization effect on biomass and essential oil of lavender (*Lavandula dentata* L.). Ind. Crops Prod..

[52] Kivrak S. (2018). Essential oil composition and antioxidant activities of eight cultivars of lavender and lavandin from western Anatolia. Ind. Crops Prod..

[53] Marincas O., Feher I. (2018). A new cost-effective approach for lavender essential oils quality assessment. Ind. Crops Prod..

[54] Dobreva A., Petkova N., Todorova M., Gerdzhikova M., Zherkova Z., Grozeva N. (2024). Organic vs. conventional farming of lavender: Effect on yield, phytochemicals and essential oil composition. Agronomy.

[55] Caprari C., Fantasma F., Divino F., Bucci A., Iorizzi M., Naclerio G., Ranalli G., Saviano G. (2021). Chemical Profile, In vitro biological activity and comparison of essential oils from fresh and dried flowers of *Lavandula angustifolia* L.. Molecules.

[56] Costea T., Străinu A.M., Gîrd C.E. (2019). Botanical characterization, chemical composition and antioxidant activity of Romanian lavander (*Lavandula angustifolia* Mill.) flowers. Studia Universitatis “Vasile Goldiş”. Ser. Ştiinţele Vieţii..

[57] Ognyanov I. (1983). Bulgarian lavender and Bulgarian lavender oil. Perf. Flavor..

[58] Bogdan M., Bungau S., Tit D.M., Copolovici L., Behl T., Otrisal P., Aleya L., Cioca G., Berescu D., Uivarosan D. (2020). Variations in the chemical composition of the essential oil of *Lavandula angustifolia* Mill., Moldoveanca 4 Romanian variety. Rev. Chim..

[59] Verma R.S., Rahman L.U., Chanotiya C.S., Verma R.K., Chauhan A., Yadav A., Singh A., Yadav A.K. (2010). Essential oil composition of *Lavandula angustifolia* Mill. cultivated in the mid hills of Uttarakhand, India. J. Serb. Chem. Soc..

[60] Baydar H., Kineci S. (2009). Scent composition of essential oil, concrete, absolute and hydrosol from Lavandin (*Lavandula x intermedia* Emeric ex Loisel.). J. Essen. Oil Bear. Plants.

[61] (2002). Oil of Lavender (Lavandula angustifolia Mill.).

[62] (2025). Essential Oil of lavandin Grosso (Lavandula x intermedia Emeric ex Loisel. “grosso”) (ex Lavandula angustifolia Mill. × Lavandula latifolia Medik. “grosso”).

[63] Gavrić T., Gadžo D., Hafner-Vuk K., Erhatić R. (2023). Antioxidant capacity and composition of essential oil of lavender and lavandin growing in Bosnia and Herzegovina. Agric. For..

[64] Sariri R., Seifzadeh S., Sajedi R.H. (2009). Anti-tyrosinase and antioxidant activity of *Lavandula* sp. extracts. Pharmacol. Online.

[65] El Abdali Y., Agour A., Allali A., Bourhia M., El Moussaoui A., Eloutassi N., Salamatullah A.M., Alzahrani A., Ouahmane L., Aboul-Soud M.A.M. (2022). *Lavandula dentata* L.: Phytochemical analysis, antioxidant, antifungal and insecticidal activities of its essential oil. Plants.

